# A Small Molecule Inhibitor Partitions Two Distinct Pathways for Trafficking of Tonoplast Intrinsic Proteins in Arabidopsis

**DOI:** 10.1371/journal.pone.0044735

**Published:** 2012-09-05

**Authors:** Efrain E. Rivera-Serrano, Maria F. Rodriguez-Welsh, Glenn R. Hicks, Marcela Rojas-Pierce

**Affiliations:** 1 Department of Plant Biology, North Carolina State University, Raleigh, North Carolina, United States of America; 2 Department of Botany and Plant Sciences, University of California Riverside, Riverside, California, United States of America; 3 Center for Plant Cell Biology, University of California Riverside, Riverside, California, United States of America; Ghent University, Belgium

## Abstract

Tonoplast intrinsic proteins (TIPs) facilitate the membrane transport of water and other small molecules across the plant vacuolar membrane, and members of this family are expressed in specific developmental stages and tissue types. Delivery of TIP proteins to the tonoplast is thought to occur by vesicle–mediated traffic from the endoplasmic reticulum to the vacuole, and at least two pathways have been proposed, one that is Golgi-dependent and another that is Golgi-independent. However, the mechanisms for trafficking of vacuolar membrane proteins to the tonoplast remain poorly understood. Here we describe a chemical genetic approach to unravel the mechanisms of TIP protein targeting to the vacuole in Arabidopsis seedlings. We show that members of the TIP family are targeted to the vacuole via at least two distinct pathways, and we characterize the bioactivity of a novel inhibitor that can differentiate between them. We demonstrate that, unlike for TIP1;1, trafficking of markers for TIP3;1 and TIP2;1 is insensitive to Brefeldin A in Arabidopsis hypocotyls. Using a chemical inhibitor that may target this BFA-insensitive pathway for membrane proteins, we show that inhibition of this pathway results in impaired root hair growth and enhanced vacuolar targeting of the auxin efflux carrier PIN2 in the dark. Our results indicate that the vacuolar targeting of PIN2 and the BFA-insensitive pathway for tonoplast proteins may be mediated in part by common mechanisms.

## Introduction

The vacuole is an essential and dynamic organelle in plant cells with critical roles in storage of proteins, ions and metabolites and maintaining cellular turgor, and homeostasis [1]. The activity of vacuole membrane proteins is important for plant responses to multiple environmental stresses and has implications on agricultural systems [1]. Two types of vacuoles have been described in plants, a lytic vacuole and a protein storage vacuole. The lytic vacuole has acidic pH, is abundant in mature tissues, and is homologous to the animal lysosome. The protein storage vacuole (PSV) has a neutral pH and is the main protein storage compartment in developing seeds. In barley, pea and Arabidopsis root tips, the lytic vacuole is marked by the presence of the Tonoplast Intrinsic Protein1;1 (TIP1;1/γTIP) and proteases such as the cysteine protease Aleurain, whereas the protein storage vacuole is labeled with TIP3;1 (αTIP) and proteins of the globulin group such as barley lectin [2], [3]. However, the biogenesis of each type of vacuole is rather complex and their identity and protein content may be dependent on the plant species, developmental stage, and cell types analyzed [4], [5], [6], [7], [8], [9], [10]. In tobacco root tips, the mechanisms of vacuole biogenesis are cell-type specific, and lytic vacuoles are generated by fusion and maturation of PSVs [9]. In Arabidopsis, lytic and protein storage vacuoles are found in different developmental stages but have not been detected as independent compartments in a single cell [4], [7], [8]. Intriguingly, in hypocotyls of Arabidopsis germinating seedlings, lytic sub-compartments were observed inside PSVs and these may mature into lytic vacuoles [11]. The essential nature of plant vacuoles and the multitude of species, cell types and experimental approaches utilized to characterize plant vacuoles have prevented the establishment of a unified model for vacuole biogenesis.

Vacuole biogenesis, integrity and function depend on the targeting of membrane proteins to this organelle [12], [13], [14]. The transport of membrane proteins to the vacuole is thought to occur by vesicle trafficking from the endoplasmic reticulum (ER) after translocation to the ER membrane [15], [16], [17], [18]. Two pathways have been proposed for the targeting of tonoplast proteins through the endomembrane system, one that is Golgi-dependent and another that is Golgi-independent [17]. The Golgi-dependent pathway was described for a chimeric protein containing the transmembrane domain and C-terminus of pea BP-80. In tobacco protoplasts this protein was targeted via a Brefeldin A (BFA)-sensitive pathway towards a pre-vacuolar compartment (PVC). BFA is an inhibitor of Golgi-dependent traffic because it inhibits COPI coat formation and retrograde trafficking from the Golgi to the ER [19]. Consistent with evidence for Golgi post-translational modifications, the BP-80 fusion protein contained Asn-linked glycans [17]. Recent evidence suggests that there may be two Golgi-dependent targeting pathways that differ by their dependence on the adaptor protein complex AP3 [20].

Evidence for a Golgi-independent pathway was first obtained in tobacco protoplasts, when Wortmannin and BFA did not inhibit the delivery of α-TIP to the vacuole [21]. Later, it was shown that in tobacco protoplasts the C terminus of bean α-TIP was sufficient to prevent a reporter protein from entering the Golgi in its route to the vacuole [17]. In Arabidopsis protoplasts, the trafficking of a HA-TIP3;1 is insensitive to BFA treatment, the co-expression of the dominant negative form AtRab1, or the overexpression of Atsec23, all of which inhibit ER-Golgi traffic [22]. In addition, Arabidopsis TIP3;1-YFP targeting was dependent on COPII and this cargo was mis-targeted when mutant forms of Rha1, Ara6 and Rab7 were transiently overexpressed in tobacco leaf epidermal cells [23]. Similarly, the trafficking of the rice Two-Pore K^+^ b (TPKb) channel is also BFA-insensitive [24]. While there is some evidence for Golgi-independent trafficking of tonoplast proteins in Arabidopsis and tobacco, all the data thus far have come from transient expression studies and, in most cases, heterologous systems.

TIP family proteins are transport facilitators for small molecules across the vacuolar membrane. Plant TIPs have been shown to transport water, CO_2_, H_2_O_2_ and ammonium, underscoring putative roles in cell homeostasis and signaling [25], [26]. The family of TIP proteins in Arabidopsis is formed by five subgroups, TIP1/γTIP, TIP2/δTIP, TIP3/α-TIP, TIP4 and TIP5. Live-cell imaging of fluorescent protein fusions with members of the TIP1, TIP2 and TIP3 subgroups indicated that the expression patterns of these genes is developmentally regulated, and that when co-expressed in the same cell, these proteins localize to the same vacuole [4], [6]. TIP3;1 accumulates in maturing embryos and dry seeds, and it is replaced by TIP1;1 during germination [4]. TIP1 and TIP2 proteins were detected in defined tissue types in roots [8]. With the exception of TIP3;1, which traffics to the vacuole via a Golgi-independent pathway [22], the trafficking of TIP family proteins is not yet characterized. Intriguingly, TIP3;1 was recently shown to localize to both tonoplast and plasma membrane during embryo maturation and seed germination, and this dual localization appears to be specific to this isoform [27].

Maintenance of vacuolar membrane integrity is essential for plant growth and development, and yet little is known about the mechanisms regulating the trafficking of membrane proteins to the vacuole [1], [12], [28]. Unlike an extensive record for trafficking of soluble vacuolar proteins [29], [30], [31], [32], [33], only a few endomembrane components, including the SNARE protein SYP21, and three Rab proteins, Rha1, Ara6 and Rab7, have been implicated in tonoplast membrane trafficking [23], [34]. In this report, we took advantage of the model plant Arabidopsis and a chemical genetic approach to analyze the trafficking of the TIP protein family in stably transformed plants. We report a new set of inhibitors of tonoplast protein trafficking in Arabidopsis with diverse effects on few or multiple endomembrane trafficking pathways. By characterizing the bioactivity of one of these compounds, we provide evidence for multiple pathways targeting the TIP family of proteins to the vacuole. We show that GFP-TIP2;1 and TIP3;1-YFP, but not TIP1;1-YFP, travel in a BFA-insensitive pathway in hypocotyls of Arabidopsis stably transformed plants. Extensive characterization of this unique inhibitor underscored a new link between the BFA-insensitive pathway for tonoplast proteins and the vacuolar targeting of PIN2.

## Materials and Methods

### Plant materials and growth conditions

The GFP-TIP2;1 (previously named GFP-δTIP) marker line was previously described [35]. A pUBQ10::mCherry-HDEL marker was generated by substituting the 35S promoter from the ER-rK marker [36] with a pUBQ10 promoter from pNIGEL vector [37] using traditional cloning techniques. This marker was introduced into the GFP-TIP2;1 marker line via Agrobacterium-mediated transformation. Other marker lines were previously described, PIP2A-GFP [35], 35S::TIP1;1-YFP, 35S::TIP3;1-YFP [4], NAG1-GFP [38], VHA-a1-YFP, [39], TT12-GFP [40], SNX1-GFP [41], YFP fusions to SYP32, VAMP711, Rab G3f, Rab A5d and Rab C1 [37], Aleu-GFP [42], PIN1-GFP [43], PIN2::PIN2-GFP [44], PIN3-GFP [45], PIN4-GFP [46] and PIN7-GFP [47]. Plants were incubated in a growth chamber at 22°C under a 16 h light photoperiod.

### Microscopy

A Zeiss LSM 710 confocal microscope from the Cellular and Molecular Imaging Facility at North Carolina State University was used. A Leica 40X water objective (1.1 NA) was used for all experiments. Simultaneous acquisition in channel mode and two Main Dichroic Beam Splitters (MBS) for 488 and 561 nm were used to image the double marker line. The pinhole was maintained at 1 AU (41 μm). GFP was excited at 488 nm and emission was collected at 492–570 nm. mCherry was excited at 561 and emission was collected at 588–696 nm. YFP was excited at 488 nm and detected in the 492 to 557 nm range. All experiments described here were carried out at least three times with similar results. Root morphology and root hairs were imaged either in a Leica stereomicroscope or a Leica DM5000 compound microscope equipped with a Leica digital camera.

### Chemical screen and treatments

The chemical library of 360 pollen growth inhibitors was described before [48] and was maintained at 5 mg/ml in 100% DMSO stock. Primary screens were carried out in 200 μl of AGM (0.5X MS, 3g/L GelRite, 1 % sucrose) with 3-day-old plants containing 1 μl of the library stock (25 μg/ml). Fluorescent marker localization was analyzed in at least three seedlings per compound after 48 h. Seventy-six hits were identified in a primary screen and 36 passed a secondary screen using three concentrations (∼1 µM, 10 μM and 100 μM). After one more round of selection, only 16 had reproducible effects. Only 5 compounds were confirmed as hits after new stocks from ChemBridge were tested.

Unless specified, seedling treatments were done by transferring 3-day-old plants to AGM media supplemented with chemical and incubation in the light for 48 h. PIN markers were exposed for 18 h. All treatments in the light or dark with C834 were carried out on plates that were pre-incubated in the light for 16–20 h. BFA was from Sigma.

## Results

### Identification of inhibitors of tonoplast protein trafficking

**Figure 1:**
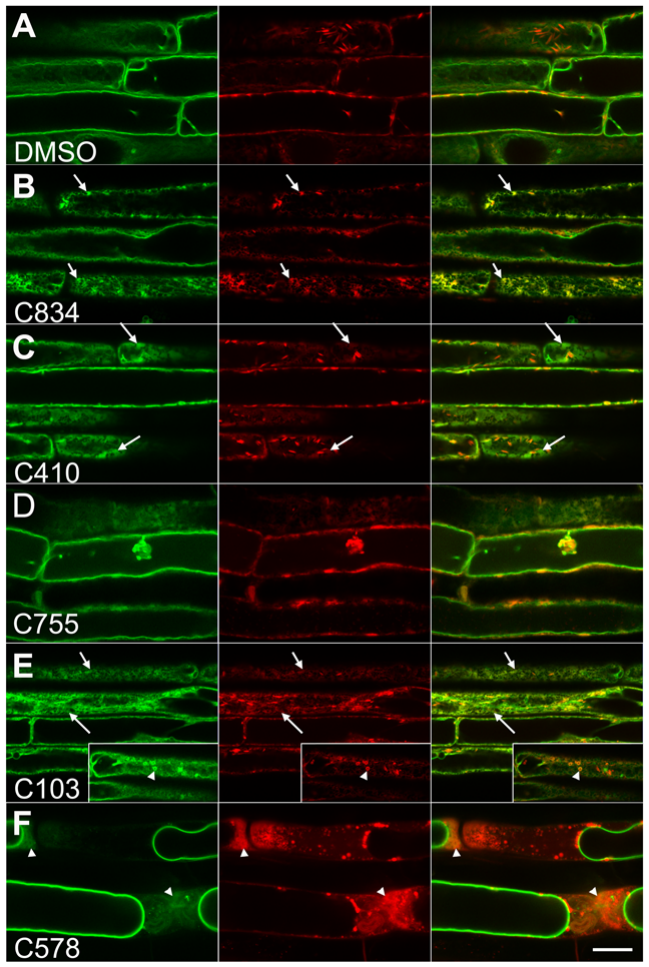
Chemical hits induce the accumulation of GFP-TIP2;1 to an ER-like network or aberrant compartments. Three-day-old seedlings expressing GFP-TIP2;1 and mCherry-HDEL were exposed to DMSO (control, A), 55 uM C834 (B), 62.34 μM C410 (C), 88 μM C755 (D), 79.14 μM C103 (E) or 80 μM C578 (F) for 48 h, and imaged under a confocal microscope. Signals from GFP-TIP2;1 (green), mCherry-HDEL (red) and merged image are shown. Insets in (E) show cells with vesiculated structures. Arrows indicate sites of co-localization at the ER network. Arrowheads indicate vesiculated ER structures (E) or cytoplasmic staining (F). Bar  = 20 μm.

**Figure 2:**
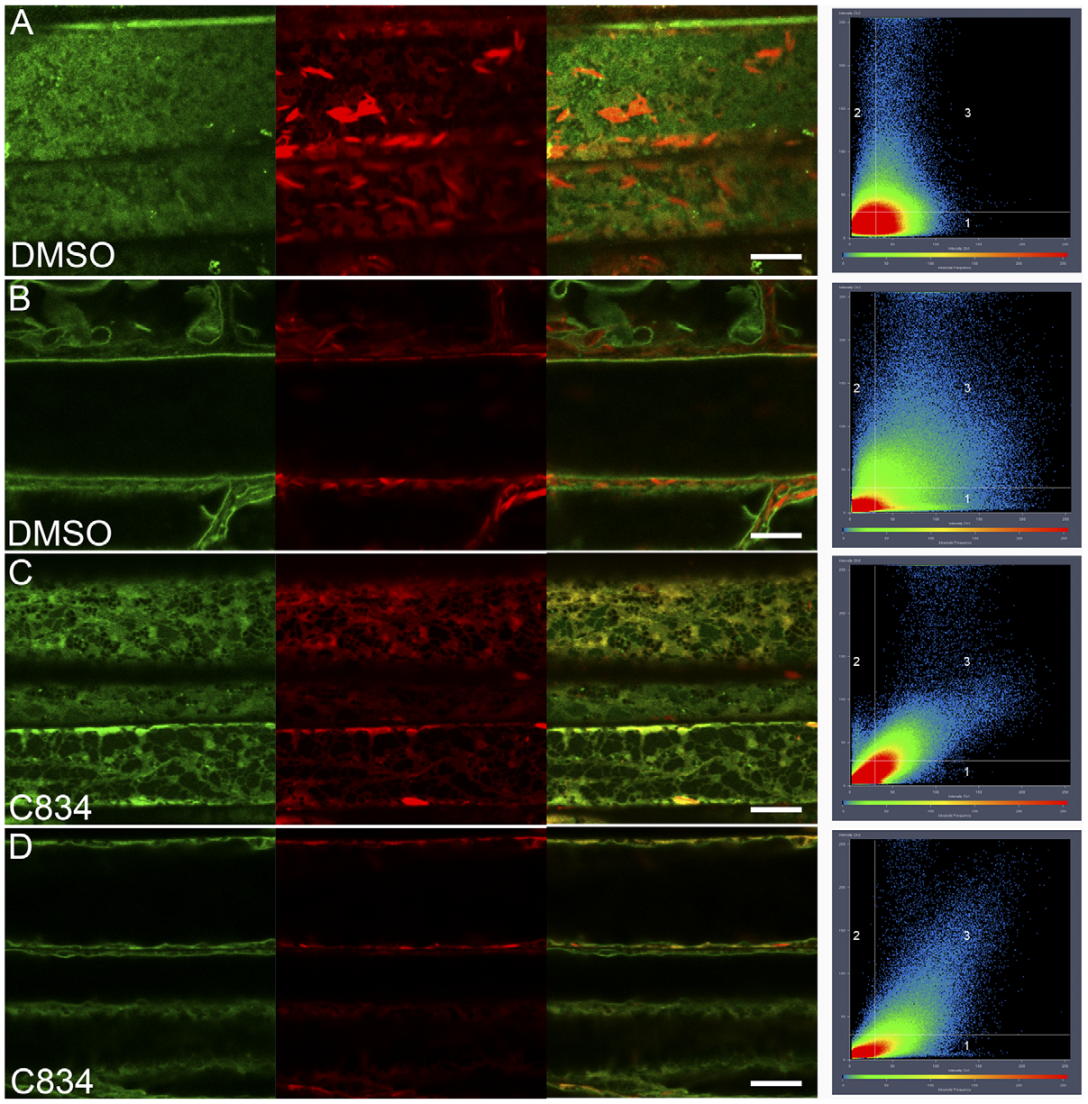
GFP-TIP2;1 accumulates in the Endoplasmic Reticulum in C834-treated cells. Three-day-old seedlings expressing GFP-TIP2;1 and mCherry-HDEL were exposed to DMSO (control, A, B) or 55 uM C834 (C, D) for 48 h, and imaged under a confocal microscope. Signals from GFP-TIP2;1 (green), mCherry-HDEL (red) and merged image are shown. Scatter plots showing pixel intensity in the red (Y axis) or green (X axis) channel for each image is also shown. While signal in the DMSO treated cells are distributed equally (A, B), the pixels in the C834-treated cells show strong correlation between the two channels (C, D). Both cortical (A, C) and medial (B, D) sections of the same cells are shown. Bar  = 10 μm.

With the goal of characterizing membrane protein trafficking to the vacuole in Arabidopsis, we used a chemical genetic approach to identify compounds that inhibited tonoplast membrane trafficking. We designed a screen using plants that carry the GFP-TIP2;1 fusion protein as the tonoplast marker [35] and the mCherry-HDEL construct as a marker for the ER [36]. Under normal conditions, the two fluorescent markers do not co-localize by confocal microscopy because they occupy independent compartments (Figure 1A). Controls using lines with single markers demonstrate that there is no bleed-though between GFP and mCherry signals under our microscope settings (Figure S1). We predicted that conditions that disturbed the trafficking of a tonoplast protein would result in its accumulation at intermediate compartments including the ER, and full or partial co-localization of the two markers. A library of 360 pollen-growth inhibitors previously identified in a screen of ∼48,000 compounds [48] was used for the screen. Given the important role of the endomembrane system for the elongation of pollen tubes [49], it is not surprising that chemicals that perturb endocytosis and the recycling of plasma membrane proteins have already been identified from this library [48], [50], [51]. Only 5 out of the 360 compounds passed the screen as they induced mis-localization of the GFP-TIP2;1 marker (Table S1). Most compounds resulted in the localization of the GFP-TIP2;1 marker in a compartment that resembles the cortical ER network as predicted (Figure 1 B–E). However, C755 induced the accumulation of GFP-TIP2;1 in the ER-network as well as other aggregated structures (Figure 1D). Two compounds induced more severe phenotypes and may represent broad inhibitors of the endomembrane system: C103 induced the accumulation of GFP-TIP2;1 in the ER network as well as in small vesicular structures that were also labeled with the ER marker (Figure 1E, inset). C578-treated plants showed GFP-TIP2;1 localization in tonoplast, but also in diffused cytoplasmic structures (Figure 1F). C103 and C578 may affect the biogenesis of the ER as indicated by the appearance of the mCherry-HDEL marker (Figure 1E, F). Overall, the diverse phenotypes of chemical treated plants suggest that the drugs affect independent targets and should be valuable tools to dissect the trafficking of membrane proteins to the vacuole. To confirm that the structures observed in C834-treated cells (Figure 1B) corresponded to ER, we acquired Z optical sections for DMSO and C834-treated plants expressing the GFP-TIP2;1 and mCherry-HDEL markers (Figure 2, Movie S1, Movie S2). Cortical sections of GFP-TIP2;1 appear as a smooth surface with diffuse signal that does not align well with the mCherry-HDEL localized network (Figure 2A). In medial sections of the control, GFP-TIP2;1 localization is typical of a defined tonoplast membrane with constant intensity along the boundary of the vacuole (Figure 2B). In contrast, clear network structures that co-localize with the ER marker are visible in GFP-TIP2;1 in cortical sections of the C834-treated cells (Figure 2C). In medial sections, the GFP-TIP2;1 signal is discontinuous along the periphery of the vacuole, and this signal co-localizes significantly with the ER marker (Figure 2D). Scatter plots from these images demonstrate the two markers co-localize in the C834 treatment but not in the DMSO control (Figure 2, Figure S2).

In order to establish the specificity of the chemical inhibitors, we exposed a series of fluorescent markers to each of the hit compounds. These included NAG1-GFP as a marker for Golgi [38], VHA-a1-YFP as a marker for the *trans*-Golgi network [39], and PIP2A-GFP as marker for plasma membrane [35]. As shown in Figure S3, neither C834 nor C755 had any effect on these membrane proteins. However, C410 and C103 inhibited the trafficking of the plasma membrane marker PIP2A-GFP, as this marker accumulated in an ER pattern towards the upper most parts of the root. As indicated before, C578 is a non-specific inhibitor of the endomembrane system because it induced the mis-localization of Golgi and TGN markers to an undefined cytosolic body (Figure S3Q, R) similar to the one observed for GFP-TIP2;1 and mCherry-HDEL (Figure 1F).

### C834 bioactivity uncouples two pathways for tonoplast protein trafficking

**Figure 3:**
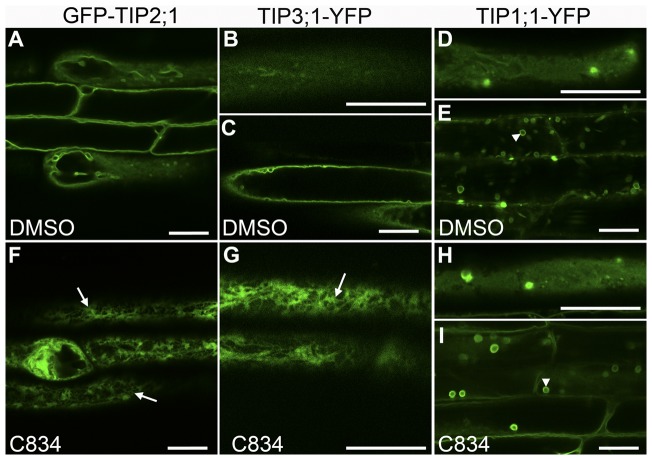
C834 uncouples two pathways for tonoplast proteins trafficking. Three-day-old seedlings expressing GFP-TIP2;1 (A, F), TIP3;1-YFP (B, C, G) or TIP1;1-YFP (D, E, H, I) were exposed to DMSO (control, A–E) or 55 μM C834 (F–I) for 48 h and imaged under a confocal microscope. ER network localization (arrows) was observed in C834-treated GFP-TIP2;1 and TIP3;1-YFP seedlings, but not in TIP1;1-YFP. Cortical sections are shown in B, D and F-H. Medial sections are shown in A, C, E and I. Arrowheads indicate vacuolar “bulbs” labeled with TIP1;1-YFP. Bar  = 20 μm.

We used the chemical hits to test the hypothesis that tonoplast intrinsic proteins are targeted to the vacuole by multiple pathways in root cells. Chemicals that inhibited the targeting of some but not all of the tonoplast markers provide strong evidence for a multiplicity of pathways. All the GFP-TIP2;1 trafficking inhibitors were tested for effects on the lytic vacuole marker TIP1;1-YFP and the PSV marker TIP3;1-YFP in Arabidopsis roots [4]. In the DMSO control, GFP-TIP2;1 and TIP3;1-YFP accumulated in the vacuolar membrane and they could be visualized as a smooth surface in cortical sections of the cell or as continuous membrane in medial sections (Figure 3A–C). The localization of TIP1;1-YFP was stronger in vacuolar bulbs, but it could still be detected in the tonoplast as expected (Figure 3D, E). When plants were treated with C834, both GFP-TIP2;1 and TIP3;1-YFP accumulated in the ER network (Figure 3F, G), whereas TIP1;1-YFP was found in the tonoplast and vacuolar bulbs in the same patterns as the control (Figure 3H, I). Because C834 affected trafficking of both TIP3;1 and GFP-TIP2;1 markers, but had no effect on TIP1;1, it was defined as Class I (Table 1, Figure 3). C410 and C755 induced the accumulation of all three tonoplast markers to the ER network and were defined as Class II (Table 1, Figure S4). C103 and C578 affected all three markers (Figure S4), and also disrupted the appearance of the ER marker by either forming vesiculated structures (Figure 1E) or inducing bright cytoplasmic staining (Figure 1F). These two probes were defined as Class III (Table 1), and they represent broader inhibitors of the endomembrane system compared to Class I and II. Our finding that the Class I compound C834 affected GFP-TIP2;1 and TIP3;1-YFP, but not TIP1;1-YFP indicated that the former are trafficked via similar mechanisms, but the latter utilizes a different pathway that is insensitive to C834. Here, we describe the bioactivity of C834 in more detail (Table S1).

**Table 1:** Effects of chemical hits on three TIP proteins and mCherry-HDEL.

Class	Compound	GFP-TIP2;1	TIP3;1-YFP	TIP1;1-YFP	mCherry-HDEL
I	C834	ER Network	ER Network	Not affected	Not affected
II	C410	ER Network	ER Network	ER Network	Not affected
II	C755	ER Network, aggregates	ER Network	ER Network	Not affected
III	C103	ER Network, “vesiculated” structures	ER Network, “vesiculated” ER structures	ER Network, “vesiculated” structures	Vesiculated
III	C578	Tonoplast, Cytoplasmic staining	Tonoplast, Cytoplasmic staining	Tonoplast, Cytoplasmic staining;	Cytoplasmic staining

### Multiple trafficking pathways are insensitive to C834

In order to understand the bioactivity of C834, we first determined the minimum concentration and incubation time for bioactivity, as well as its reversibility and its effect on plant growth. It was determined that C834 induced the accumulation of GFP-TIP2;1 at the ER as early as 8 h after treatment with 55 μM in roots, but no effects were observed in cotyledons or hypocotyls even when plants were treated at 110 μM for 48 h. Possible reasons for this result are that a C834-target is not expressed in shoots or that its inactivation is overcome by the expression of a functionally redundant protein. The effects of C834 on trafficking in root cells were reversible after wash-out (Figure S5A–C), indicating that the C834 target was not irreversibly modified. We noticed early on that C834 inhibited trafficking only after the media had been incubated in the light (Figure S5D-F). Therefore, all the experiments presented here were done with light-activated C834 media. In order to determine the specificity of the trafficking pathways affected by C834 in more detail, we tested the effect of this inhibitor on the localization of multiple post-Golgi markers. First, we tested the effect of C834 on other tonoplast proteins including TRANSPARENT TESTA12 (TT12)-GFP [40] and VAMP711-YFP [37]. Neither TT12-GFP nor VAMP711-YFP were affected by C834 (Figure S6), indicating that these proteins are likely to be trafficked via a similar pathway as TIP1;1. Markers to other compartments were tested to determine the effect of the inhibitor on the endomembrane system as a whole. These represented Aleu-GFP for vacuole lumen [42], SYP32-YFP as marker for Golgi [37], SNX1-GFP as marker of the PVC/MVB [41], and YFP fusions to the endosomal markers RabG3f, RabA5d, and RabC1 [37]. Using this approach, we did not detect any differences in the localization of any of these markers between the DMSO and C834 treatments (Figure S6) indicating that, at least for the markers analyzed, the effect of C834 on trafficking was specific to a subset of tonoplast membrane proteins.

**Figure 4:**
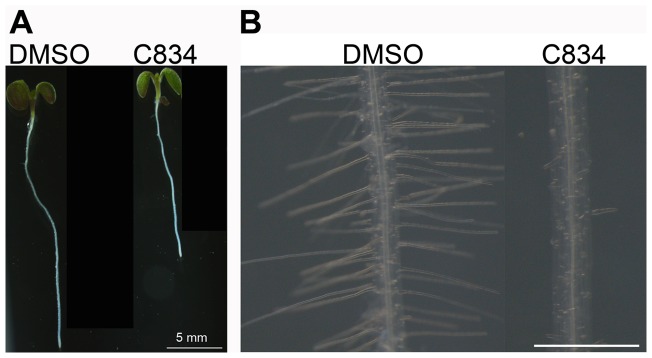
C834 is a strong inhibitor of root hair elongation. Plants were grown in the light in media containing DMSO or 55 μM C834 for 7 days prior to imaging. Bar  = 5 mm (A) or 1 mm (B).

Plants grown in the light in the presence of C834 showed a significant (∼40%) reduction in primary root elongation when compared to the control, but had normal seedling phenotype (Figure 4A). Eight-day old wild type plants grown in media containing DMSO (control) had roots of 1.7+/−0.07 cm, but those grown in the presence of 55 μM C834 were 1.08+/−0.07 cm. Root hair elongation was dramatically suppressed by C834 treatment (Figure 4B, C), possibly due to the critical roles of the endomembrane trafficking on root hair growth [52]. Overall, these results indicate that C834 had mild effects on seedling development, except for root and root hair elongation, which were dramatically affected.

### TIP2;1 and TIP3;1 traffic to the tonoplast via a BFA-insensitive pathway

**Figure 5:**
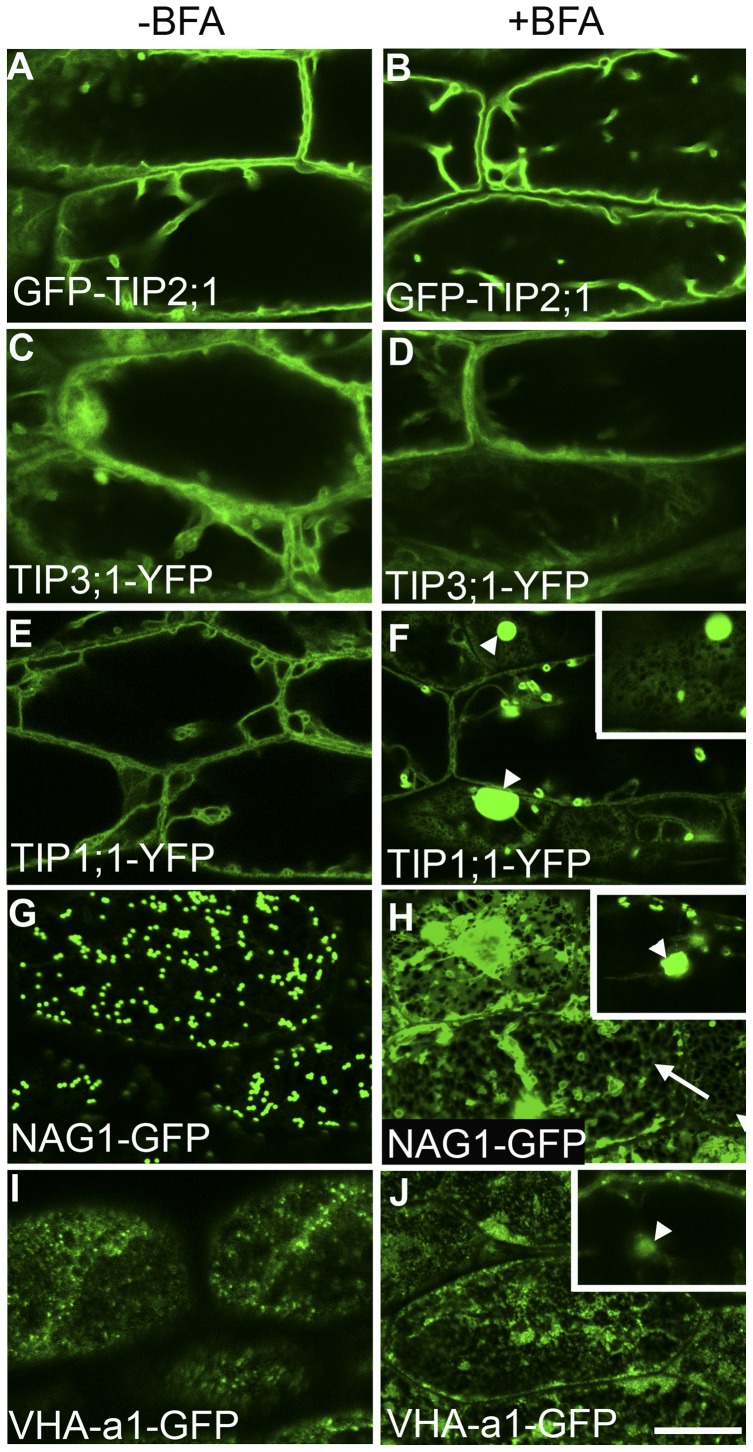
Tonoplast trafficking of TIP2;1 and TIP3;1 is insensitive to BFA. Three-day old seedlings expressing GFP-TIP2;1 (A, B), TIP3;1-YFP (C, D), TIP1;1-YFP (E, F), NAG1-GFP (G, H) and VHA-a1-GFP (I, J) were incubated in the presence of DMSO (-BFA, A, C, E, G, I) or 75 μM BFA (+BFA, B, D, F, H, J) for 3 h. Hypocotyl cells from treated plants were imaged by confocal microscopy. ER network localization is shown in the inset (F) or indicated with arrows (H). Internal BFA compartments labeled with TIP1;1-YFP (F), NAG1-GFP (H inset) and VHA-a1-GFP (J inset) are indicated with arrowheads. Bar  = 20 μm.

The trafficking of TIP3;1 in tobacco and Arabidopsis leaf protoplasts occurs via a Golgi-independent mechanism and is BFA insensitive [17], [22]. We hypothesized that GFP-TIP2;1 also trafficked to the vacuole via a BFA-insensitive pathway, which we propose is the target of C834, but TIP1;1 utilized a BFA-sensitive, C834-insensitive pathway similar to the one described for a BP-80 chimeric protein [17]. The fact that neither TIP3;1 [22] or GFP-TIP2;1 (data not shown) are glycosylated in Arabidopsis prevented us from examining the presence or absence of Golgi-dependent glycosylation in these proteins. As an alternative, we tested this hypothesis by examining the effects of BFA, an inhibitor of ER-to-Golgi vesicle trafficking [53], on TIP protein localization. Given the presence of BFA-resistant GNL1 in Arabidopsis root cells [54], which reduces the effects of this inhibitor on Golgi morphology [53], we could not carry out this experiment in roots. Instead, we focused on hypocotyl epidermal cells because it was previously shown that in these cells, BFA treatment results in the loss of Golgi stacks which remorph into undefined clusters or aggregates of vesicles [53], and therefore a Golgi-dependent pathway should be affected in these cells. In the absence of BFA, all three TIPs localized to the tonoplast of the central vacuole in a similar pattern as previously described (Figure 4A, C, E) [4]. In seedlings treated with BFA, we could not detect any differences on the localization of GFP-TIP2;1 or TIP3;1-YFP, which is consistent with these proteins being trafficked via a BFA-insensitive pathway (Figure 5B, D). In contrast, BFA treatments resulted in the accumulation of the TIP1;1-YFP in large and bright aggregated structures as well as the cortical ER network which were not observed in the control (Figure 5F). These BFA aggregates of TIP1;1-YFP are different in intensity to bulbs, variable in size, and do not have a defined delimiting membrane. While these large aggregates may correspond to BFA-induced aggregates of Golgi previously described [53], the ER-network localization of TIP1;1-YFP was unexpected. It was previously shown that Golgi stacks labeled with ST-GFP did not reabsorb into the ER in the presence of BFA in Arabidopsis hypocotyl epidermal cells [53]. To clarify this apparent discrepancy, we analyzed the localization of the Golgi marker NAG1-GFP after treatment with BFA. Without BFA, this marker localized to typical Golgi structures (Figure 5G). After BFA treatment, the NAG1-GFP marker was mis-localized to both the ER network and BFA compartments (Figure 5H), indicating that in Arabidopsis hypocotyls, some Golgi stacks may indeed reabsorb into the ER, and that the ER localization of TIP1;1-YFP was the result of this effect. To further characterize the BFA sensitivity of hypocotyls, we tested its effects on VHA-a1-GFP, a marker to the *trans*-Golgi network (Figure 5I) [39]. In BFA-treated plants, VHA-a1-GFP was also found in the large internal aggregates, but it did not localize to an ER network-like structure. Instead, in cortical sections of these cells, this marker was detected in diffuse aggregates or clusters of TGN membranes (Figure 5J). Therefore, the BFA compartments in hypocotyls in Arabidopsis may contain Golgi, TGN and ER proteins, but some Golgi stacks may also reabsorb into the ER in these cells. Our results combined indicate the existence of two distinct pathways for the trafficking of tonoplast proteins that differ by their sensitivity to BFA. While TIP1;1 is trafficked via a BFA-sensitive pathway, both TIP2;1 and TIP3;1 do so by a BFA-insensitive route. Overall, our results provide the first *in planta* evidence for these two independent pathways for tonoplast proteins in Arabidopsis. These results also highlight the specificity of C834 as a putative inhibitor of the BFA-insensitive pathway.

### C834 defines a link between the BFA-insensitive pathway and PIN2 vacuolar targeting

**Figure 6:**
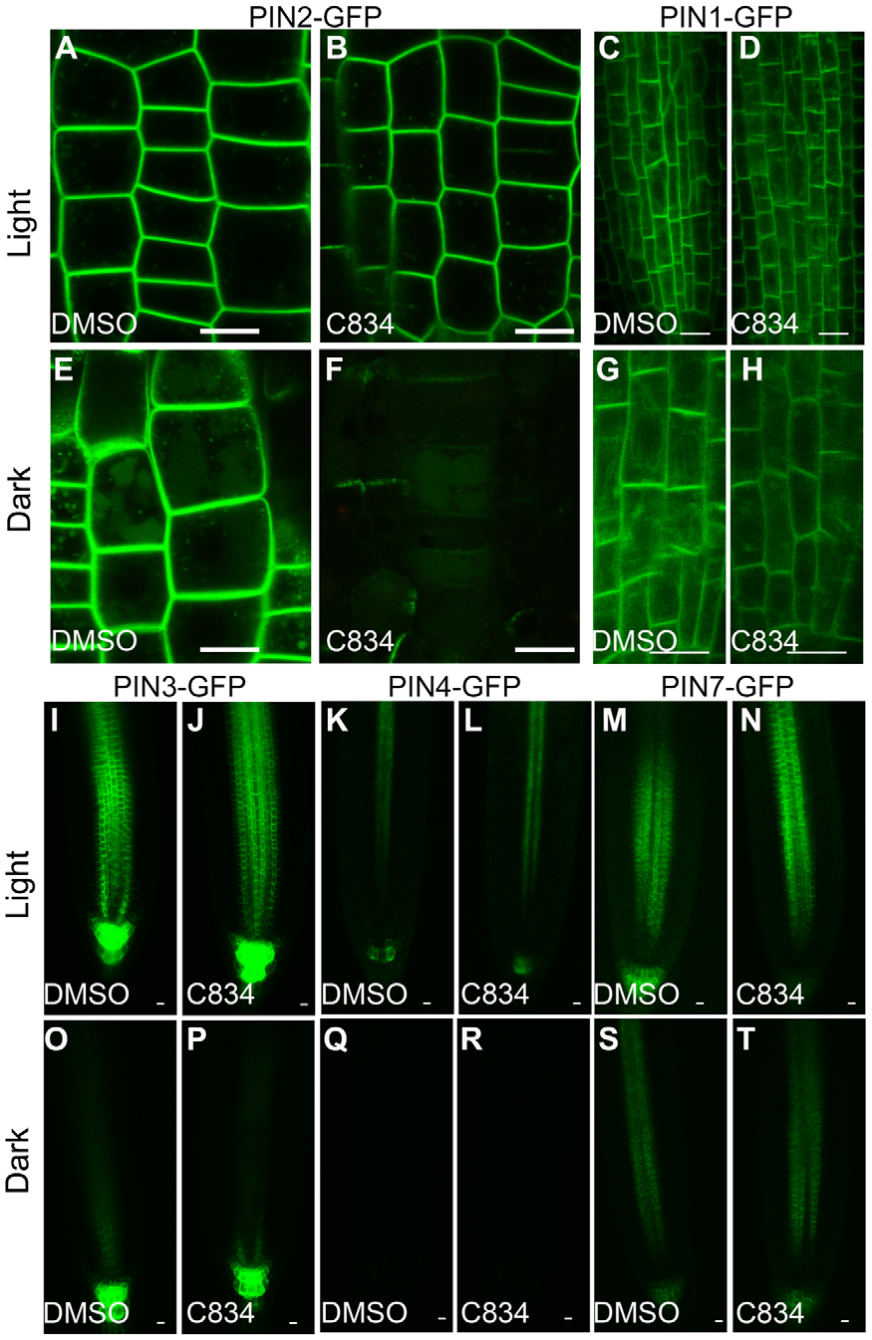
C834 enhances the vacuolar targeting and degradation of PIN2-GFP in the dark. Four-day-old seedlings expressing PIN1-GFP, PIN2-GFP, PIN3-GFP, PIN4-GFP or PIN7-GFP were transferred to either DMSO or 55 μM C834 for 18 h in the light (A–D, I–N) or the dark (E–H, O–T). All images of each marker were taken at the same microscope settings. Bar  = 10 μm.

Proteins traffic to the vacuole either via a biosynthetic route such as the one used by the TIP proteins, or an endocytic pathway for plasma membrane proteins that are trafficked to the vacuole for degradation [55], [56], [57]. The PIN family of auxin efflux carriers are plasma membrane proteins that have critical roles in the polar transport of auxin. These proteins are endocytosed into the Early Endosome/TGN, where they either undergo constitutive recycling, or transported via the PVC into the vacuole [58]. Vacuole accumulation of PIN proteins can be visualized in seedlings treated with ConcanamycinA (ConcA) or in seedlings treated in the dark because both treatments enhance the stability of PIN-GFP in the vacuole lumen [59]. We therefore wanted to test whether C834 affected vacuolar targeting of PIN proteins both in the light and in the dark. C834 did not induce significant changes on the localization of PIN1-GFP, PIN2-GFP, PIN3-GFP, PIN4-GFP or PIN7-GFP markers when seedlings were incubated in the light (Figure 6A–D, I–N), indicating that C834 does not impair the trafficking of PINs or endocytic recycling in the light. When control plants were incubated in the dark, PIN2-GFP was detected at the plasma membrane as well as the central vacuole as previously reported (Figure 6E) [59]. In contrast, C834 treatment of PIN2 in the dark resulted in significant reduction of PIN2 levels at the plasma membrane (Figure 6F). This effect was specific to PIN2-GFP, because in the case of PIN1-GFP, PIN3-GFP, PIN4-GFP or PIN7-GFP there were no differences in their plasma membrane localization between control and chemically treated plants (Figure 6 G, H, O–T). We hypothesize that in the dark-treated plants, C834 enhanced the targeting of PIN2 to the vacuole in its route for degradation. Interestingly, our C834-dark treated plants showed very similar phenotypes as those of the PIN2-eGFP marker when these plants were grown in the dark for 5 days (see Figure 4 A,B in [60]). These results suggest that C834 enhances the dark-induced regulation of PIN2 vacuolar targeting.

In order to test if the synergistic effect between dark and C834 treatments was the result of overall vacuolar trafficking defects, we exposed PIN2-GFP to the Class II chemicals, C410 and C755, which disrupt tonoplast protein trafficking (Table 1). Neither of the two compounds induced significant differences on PIN2-GFP accumulation in any of the conditions tested (Figure S7). These results indicate that PIN2 vacuolar trafficking was specifically enhanced by C834 in dark-treated cells and suggested a novel link between the BFA-insensitive trafficking of tonoplast proteins to the vacuole and PIN2 vacuolar targeting.

## Discussion

We describe here a new set of chemical inhibitors of the plant endomembrane system using a confocal microscope-based screen. Most of the hits targeted all three members of the TIP protein family and induced varying degrees of phenotypes on other endomembrane markers. The diversity of phenotypes from chemical-treated plants and the lack of structural similarity between these compounds point to multiple targets being affected by these probes. The phenotypes detected in our screen also indicate that Arabidopsis is a good system to study tonoplast protein trafficking *in planta* and that trafficking inhibition can be readily observed in a manner that may be intractable with mutants.

### Reaching the vacuole via two pathways

**Figure 7:**
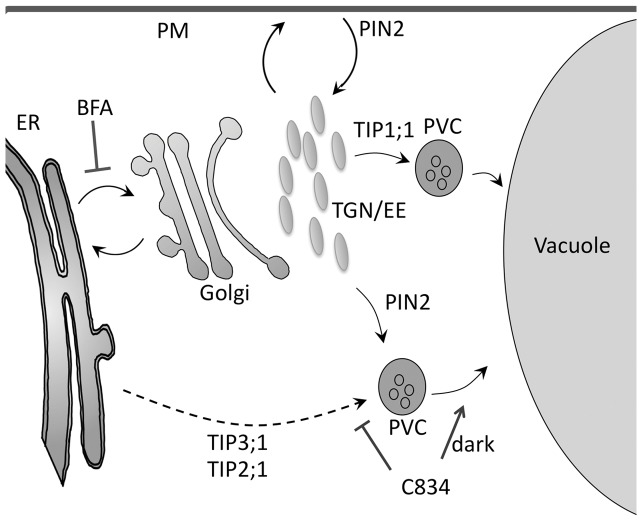
Proposed model for two pathways for TIP protein trafficking to the vacuole. A Golgi-dependent pathway may be used by TIP1;1 and is sensitive to BFA but insensitive to C834. A Golgi-independent pathway may be used by TIP3;1 and TIP2;1 and is BFA-independent and sensitive to C834. The pathway for PIN2 trafficking to the vacuole in the dark may merge with the Golgi-independent pathway at an intermediate pre-vacuolar compartment.

C834 inhibited the trafficking of GFP-TIP2;1 and TIP3;1-YFP, but not TIP1;1-YFP, which indicated the presence of two independent pathways for TIP proteins in stably transformed Arabidopsis plants. We propose that C834 is an inhibitor of the BFA-insensitive pathway for tonoplast proteins. Given the evidence that TIP3;1-YFP and GFP-TIP2;1 are targeted via a BFA-insensitive pathway in Arabidopsis hypocotyls (our results) and in protoplasts [22], and that TIP3;1 trafficking in protoplast is Golgi-independent [22], our current model is that members of the TIP family are targeted to the vacuole via either a Golgi-dependent or a Golgi-independent pathway (Figure 7). However, more experiments are necessary to determine if the BFA-insensitive pathway described here is indeed independent of the Golgi. Our results are consistent with a previous report where two distinct targeting pathways were proposed for TIP3;1 and BP-80 using a transient assay in tobacco protoplasts [17], and for a BFA-insensitive pathway for TIP3;1 trafficking in the same system [21]. Multiple pathways for membrane proteins to the vacuole were also identified in transient assays using tobacco leaf epidermis. In that system, the calcineurin binding protein CBL6 was trafficked in a COPII-independent manner, while αTIP/TIP3;1 and Vam3/SYP22 were not [23]. It is unclear if the targeting of TIP3;1 via the BFA-insensitive pathway is also COPII-dependent in Arabidopsis. While BFA treatments may be used to differentiate these two pathways as well, BFA affects all Golgi-dependent pathways and it also has major effects on endomembrane morphology and endocytosis [53], [61]. The lack of effects of C834 on the localization of at least twelve endomembrane markers suggests that C834 is a unique new tool that may be used for targeted inhibition of the BFA-insensitive pathway for tonoplast proteins in Arabidopsis roots. C834 may inhibit an important component of the endomembrane system involved in the BFA-insensitive pathway for membrane proteins. Identification of the C834 target(s) may shed light into the mechanisms of this pathway and its interactions with other trafficking pathways. However, the possibility exists that, in contrast to that of hypocotyls and protoplasts, the trafficking of the TIP3;1-YFP and GFP-TIP2;1 is Golgi-dependent in Arabidopsis roots, and in this case C834 would target a highly specific but unknown mechanism for TIP3;1 and TIP2;1 traffic to the vacuole. If this were the case, this would add one more layer of complexity to the regulation of the endomembrane system.

We cannot fully exclude the possibility that C834 acts by enhancing a retrograde traffic of tonoplast proteins from the vacuole back to the ER instead of acting as an inhibitor of anterograde traffic. However, the loss of TIP3;1-YFP and GFP-TIP2;1 from the vacuolar membrane during C834 treatments are not consistent with this possibility, as proteins that reach the ER by these means would be expected to eventually enter the anterograde pathway and reach the vacuole to some extent. More mechanistic and detailed analysis of the C834 inhibition could be used to differentiate between these possibilities, and our lab is moving in this direction.

TIPs belong to the family of major intrinsic proteins (MIPs), which are transport facilitators for small molecules across membranes. TIPs form an independent clade within the MIP family and divide into five subgroups: TIP1 to TIP5 [62]. The TIP1 and TIP3 subfamilies are closer to each other and TIP2 is the most basal group [63]. So why is TIP2;1 targeted in a similar, BFA-insensitive, pathway as TIP3;1? The C-terminal domain of bean αTIP contains critical information for the pathway [17], however, TIP2;1 is more distantly related to TIP3;1 than TIP1;1 [64]. It is possible that structural similarities exist between TIP3;1 and TIP2;1 C-terminal domains that are not apparent from the primary sequence and may be used as targeting signals. In order to start addressing this question, we attempted to determine if the BFA-insensitive pathway targeted other members of the TIP family using available protein fusions under the control of native promoters [8]. For the markers that could be tested in roots (TIP1;1-YFP, TIP2;2-YFP and TIP2;3-YFP), we found that C834 induced a sharp decrease in protein accumulation of TIP2;2 and TIP2;3, maybe due to increased protein degradation at the ER. In contrast and similarly to the overexpression line, TIP1;1-YFP showed no change in protein accumulation or localization after C834 treatment (not shown). Even though we could not definitely determine that TIP2;2 and TIP2;3 were localized to the ER under C834 treatment, the fact that the fluorescence levels of this marker were drastically reduced indicates an effect on its targeting or stability. Therefore, the possibility exists that TIP2;2 and TIP2;3 may also be trafficked via BFA-insensitive mechanisms. Since neither of the TIP proteins contain a C-terminal di-leucine motif that was recently identified as a tonoplast targeting signal in the inositol transporter INT1 [65], targeting signals for this protein family remain unknown.

### ER-Golgi traffic is BFA sensitive in hypocotyls

BFA has been used in eukaryotic cells as an inhibitor of ER-Golgi traffic because its inhibition on the activity of Golgi-localized guanine-nucleotide exchange factors (GEF) for the ADP ribosylation factor (ARF) small GTPase. The active form of ARF is essential for COPI coat formation and retrograde trafficking from the Golgi to the ER [19]. Thus, BFA treatment in yeast, animal cells and most plant cells result in redistribution of Golgi membranes in to the ER. BFA also affects plasma membrane recycling in plants due to the existence of plant specific ARF-GEFs involved in recycling of plasma membrane proteins [66]. While most eukaryotic ARF-GEFs are BFA sensitive, Arabidopsis encodes two BFA resistant ARF-GEFs, GNOM-like 1 (GNL1) which is involved in ER-Golgi traffic [32], [54] and BIG3 [67]. BFA does not induce Golgi redistribution to the ER in Arabidopsis roots, because GNL1 is active in those cells and instead, Golgi stacks (as labeled by gamma COP, and GNL1-YFP) cluster around BFA compartments labeled with the TGN markers VHA-a1 and FM4-64, while the TGN is presumably functional [68]. However, in leaf cells, typical effects of BFA on Golgi has been demonstrated indicating that a BFA-sensitive ARF-GEF is important for ER-Golgi traffic [53]. While a previous report using a N-α-2,6-sialyltransferase(ST)-GFP fusion did not show ER-Golgi traffic inhibition in hypocotyls [53], we found that some Golgi stacks do reabsorb in the ER during BFA treatment when NAG1-EGFP was used. The differences between the BFA treatments of NAG1-EGFP and ST-GFP in Arabidopsis hypocotyls may be due to compartmentalization of these markers within the Golgi stacks. ST is targeted to the *trans* side of the Golgi both in mammalian cells [69] and in Arabidopsis when it is expressed ectopically [70]. NAG1 is targeted to the *medial* Golgi in mammalian cells [71] and to an early Golgi compartment in tobacco cells, where it is required for an early N-glycosylation reaction [72]. This suggests that *cis* and *medial* Golgi stacks may be reabsorbed to the ER in Arabidopsis hypocotyls, but *trans* cisternae are not. Overall, our results indicated that a BFA-sensitive ARF-GEF is expressed in hypocotyls and regulates retrograde traffic at the Golgi compartment, and BFA can be used in Arabidopsis hypocotyls for assessing Golgi-dependent mechanisms.

### PIN2 and ER-vacuole pathway share some common mechanisms

PIN2 abundance at the plasma membrane was dramatically reduced after C834 treatment in the dark, but not in light-treated plants. The vacuolar targeting of PIN2-GFP in the dark is enhanced by an unknown mechanism that is dependent on red and far-red photoreceptors [60]. This pathway is inhibited by blue light and the activity of HY5, a transcription factor that regulates photomorphogenesis [60]. Our results suggest that C834 treatment in the dark enhanced the delivery of PIN2-GFP to the vacuole for degradation in a similar manner as a long term (5 day) dark treatment [60], and that C834 may be targeting a downstream effector of the photomorphogenic response. Interestingly, the effect of C834 in the dark was exclusive of PIN2 because this compound affected none of the other PIN markers tested. This result is consistent with previous reports indicating that PINs and other plasma membrane proteins undergo endocytosis via multiple pathways. For example, endosidin1 inhibits trafficking of PIN2 but not PIN1 and PIN7 [51]. In addition, recycling of PIN1 is dependent on GNOM, but PIN2 utilizes additional ARF GEFs [73]. However, C834 is the first chemical probe that enhances vacuolar targeting and degradation of PIN2 specifically in the dark. One interpretation of our results is that this vacuolar targeting of PIN2 in dark seedlings and the BFA-insensitive pathway of tonoplast proteins share a common compartment in route to the vacuole, perhaps a specialized pre-vacuolar compartment that sorts cargo from endosomes and Golgi-independent vesicles (Figure 7). Alternatively, a C834 target may function in the two pathways, but in two distinct compartments. Further analysis with mutants with altered sensitivity to C834 will sort between these possibilities. Taken as a whole, this unique inhibitor could be a valuable tool to characterize the complexity of PIN2 vacuolar trafficking and its interactions with the BFA-insensitive pathway and the light response.

## Supporting Information

Figure S1 **Assessment of bleed-through fluorescence for imaging of GFP-TIP2;1 and mCherry-HDEL.** Seedlings expressing either single marker GFP-TIP2;1, mCherry-HDEL, or both were imaged as indicated in materials and methods. No significant signal was detected in the red channel when GFP-TIP2;1 was expressed alone and no signal was detected in the green channel when mCherry-HDEL was expressed alone. Scale bar: 10 μm.
(TIF)

Figure S2 **GFP-TIP2;1** **co-localizes with mCherry-HDEL in C834-treated cells.** Low magnification images and corresponding scatter plots of the same treatments show that in comparison to the DMSO control (A), the effect of C834 on GFP-TIP2;1 localization is present in multiple cell files (B). Bar: 50 μm.
(TIF)

Figure S3 **Effects of bioactive hits on endomembrane markers for Golgi,** ***trans*** **-Golgi network and plasma membrane.** Three-day-old seedlings expressing PIP2A-GFP (A, D, G, J, M, P), NAG1-GFP (B, E, H, K, N, Q) and VHA-a1-GFP (C, F, I, L, O, R) were exposed to DMSO (control, A–C), 55 μM C834 (D–F), 62.34 μM C410 (G–I), 88 μM C755 (J–L), 79.14 μM C103 (M–O) or 80 μM C578 (P–R) for 48 h and imaged under a confocal microscope. ER network localization (arrows) was observed in C410 and C103-treated PIP2A-GFP. Cytoplasmic diffuse fluorescence induced by C578 is indicated with arrowheads. Bar  = 20 μm.
(TIF)

Figure S4 **Class II and Class III probes disturb the trafficking of all three tonoplast intrinsic proteins.** Three-day-old seedlings expressing, TIP3;1-YFP (A, D, G, J, M), GFP-TIP2;1 (B, E, H, K, N) or TIP1;1-YFP (C, F, I, L, O) were exposed to DMSO (control, A–C), 62.34 μM C410 (D–F), 88 μM C755 (G–I), 79.14 μM C103 (J–L) or 80 μM C578 (M–O) for 48 h and imaged under a confocal microscope. Bar  = 20 μm.
(TIF)

Figure S5 **C834 bioactivity is reversible and requires light incubation.** (**A–C**) Three-day old GFP-TIP2;1 seedlings were exposed to DMSO (A) or C834 (B) for 48 h, or exposed to C834 for 24 h and then transferred to liquid MS media for 24 h (C) before imaging. 24 h of C834 treatment induced the same phenotype as the one shown in (B). (D–F) Three-day-old GFP-TIP2;1 seedlings were transferred to media containing DMSO (D), 55 μM C834 (E) or 55 μM C834 media that was previously exposed to light for 16 h (F). Plates were incubated in the dark for 48 h before microscopic analysis. Only the light-treated C834 induces the ER localization of GFP-TIP2;1. Bar  = 20 μm.
(TIF)

Figure S6 **Effect of C834 on a diverse set of endomembrane markers.** 3-day old seedlings expressing the indicated constructs were transferred to DMSO (A, C, E, G, I, K, M, O, Q) or C834 (B, D, F, H, J, L, N, P)-containing media for 48 h. Bar  = 20 μm.
(TIF)

Figure S7 **PIN2 trafficking in the dark is insensitive to Class II probes.** Four-day-old light-grown seedlings expressing PIN2-GFP were transferred to either DMSO (control, A,B), 62.34 μM C410 (C, D), 88 μM C755 (E, F) for 18 h in the light (A, C, E) or the dark (B, D, F). All images of each marker were taken at the same microscope settings. Bar  = 10 μm.
(TIF)

Movie S1 **Z series of GFP-TIP2;1 mCherry-HDEL plants from control treatments.** Three-day-old seedlings expressing GFP-TIP2;1 and mCherry-HDEL were exposed to DMSO (control) for 48 h as in Figure 2. Signals from GFP-TIP2;1 (green), mCherry-HDEL (red) and merged image are shown from a series of optical sections every 0.5 μm.
(MOV)

Movie S2 **Z series of GFP-TIP2;1 mCherry-HDEL plants from C834 treatments.** Three-day-old seedlings expressing GFP-TIP2;1 and mCherry-HDEL were exposed to 55 μM C834 (control) for 48 h as in Figure 2. Signals from GFP-TIP2;1 (green), mCherry-HDEL (red) and merged image are shown from a series of optical sections every 0.5 μm.
(MOV)

Table S1 **Chemical structure, and Chembridge and PubChem ID numbers for the tonoplast trafficking inhibitors.** Short name in parenthesis is used in the text.
(PPT)
