# Supplementary figures and images for: A Small Molecule Inhibitor Partitions Two Distinct Pathways for Trafficking of Tonoplast Intrinsic Proteins in Arabidopsis
Source: PLoS One. 2012 Sep 5;7(9):e44735. doi: 10.1371/journal.pone.0044735 (PMC3434187; doi:10.1371/journal.pone.0044735)

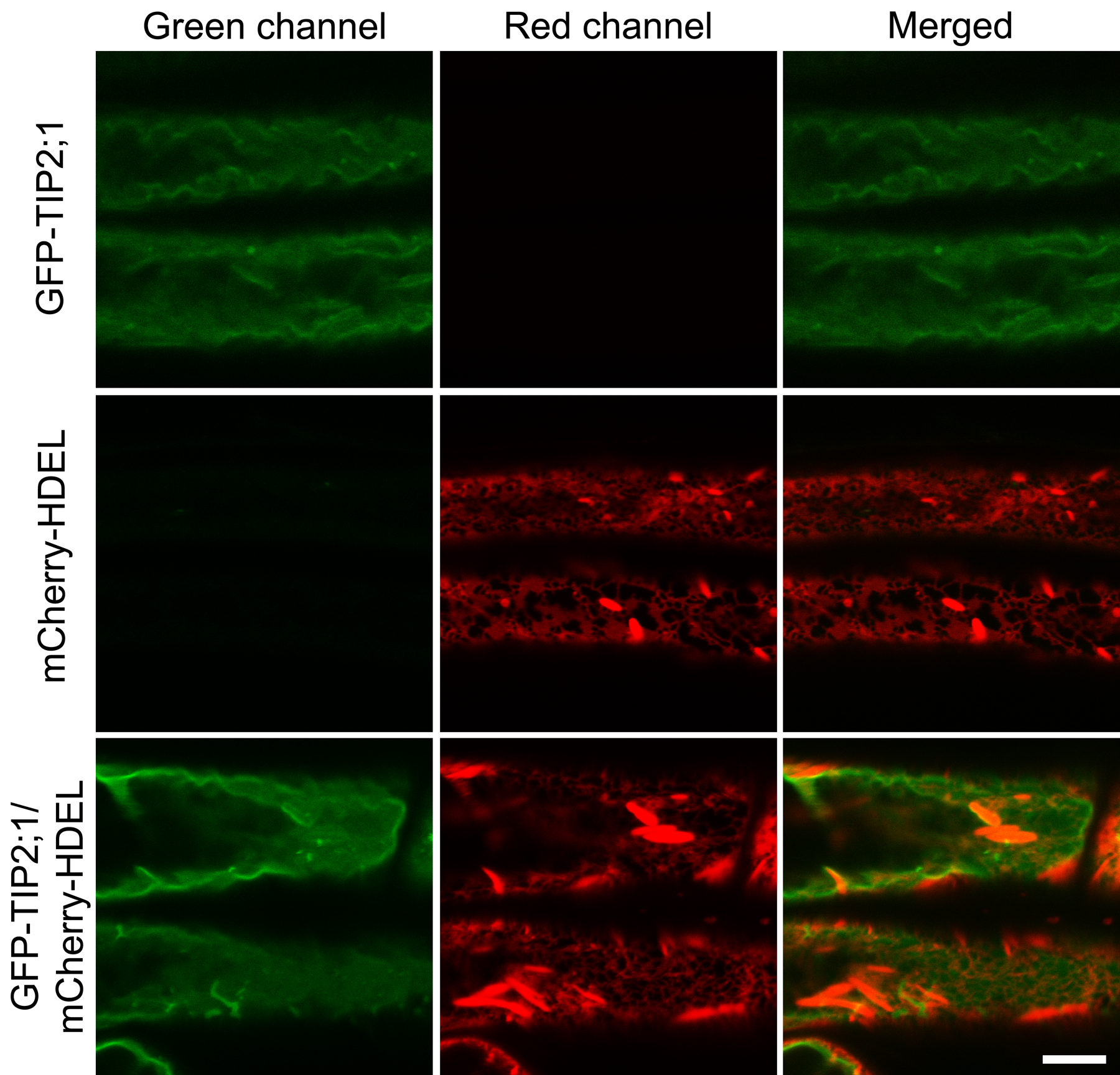

Supplement: Figure S1 — Assessment of bleed-through fluorescence for imaging of GFP-TIP2;1 and mCherry-HDEL. Seedlings expressing either single marker GFP-TIP2;1, mCherry-HDEL, or both were imaged as indicated in materials and methods. No significant signal was detected in the red channel when GFP-TIP2;1 was expressed alone and no signal was detected in the green channel when mCherry-HDEL was expressed alone. Scale bar: 10 μm. (TIF) [file pone.0044735.s001.tif]

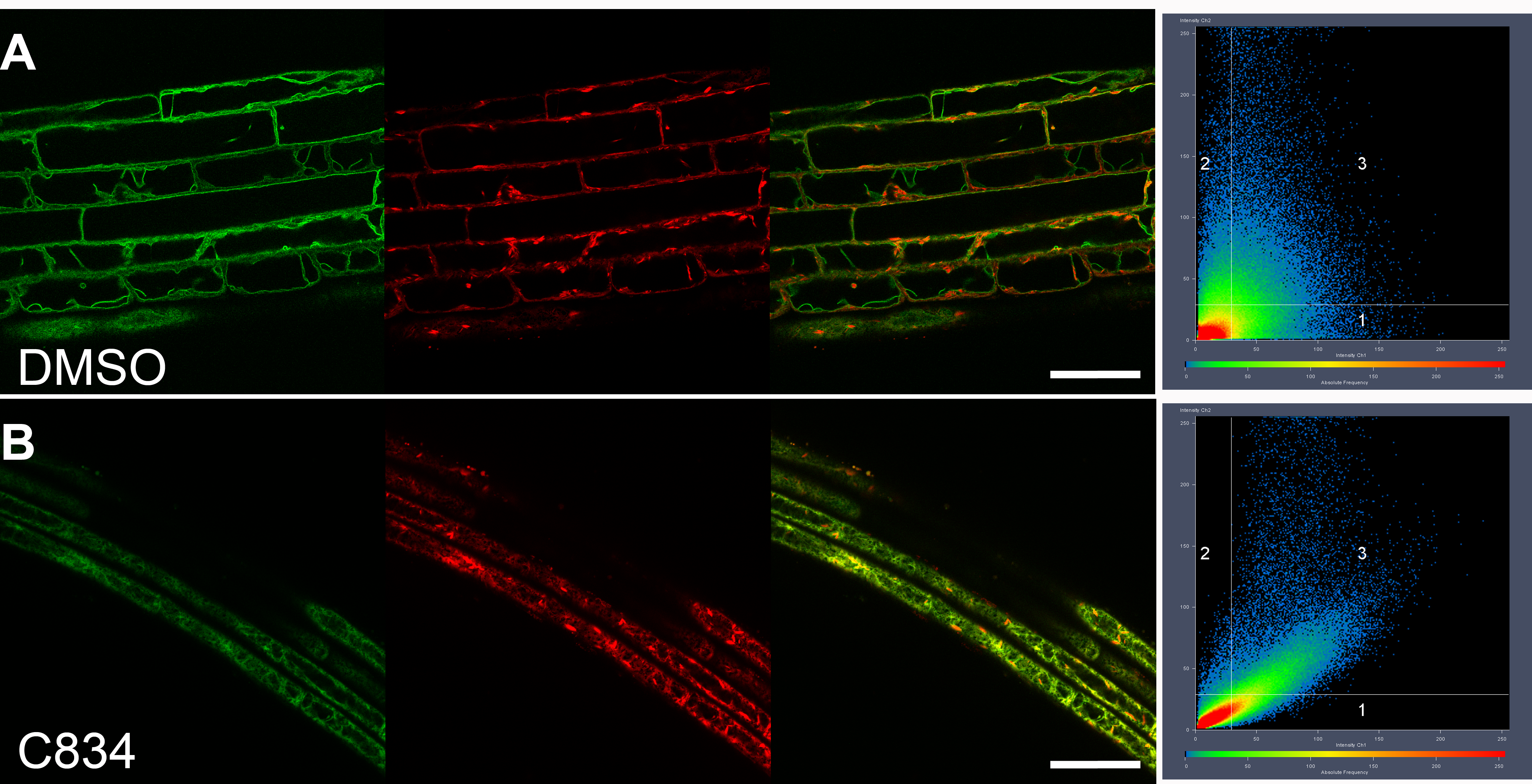

Supplement: Figure S2 — GFP-TIP2;1 co-localizes with mCherry-HDEL in C834-treated cells. Low magnification images and corresponding scatter plots of the same treatments show that in comparison to the DMSO control (A), the effect of C834 on GFP-TIP2;1 localization is present in multiple cell files (B). Bar: 50 μm. (TIF) [file pone.0044735.s002.tif]

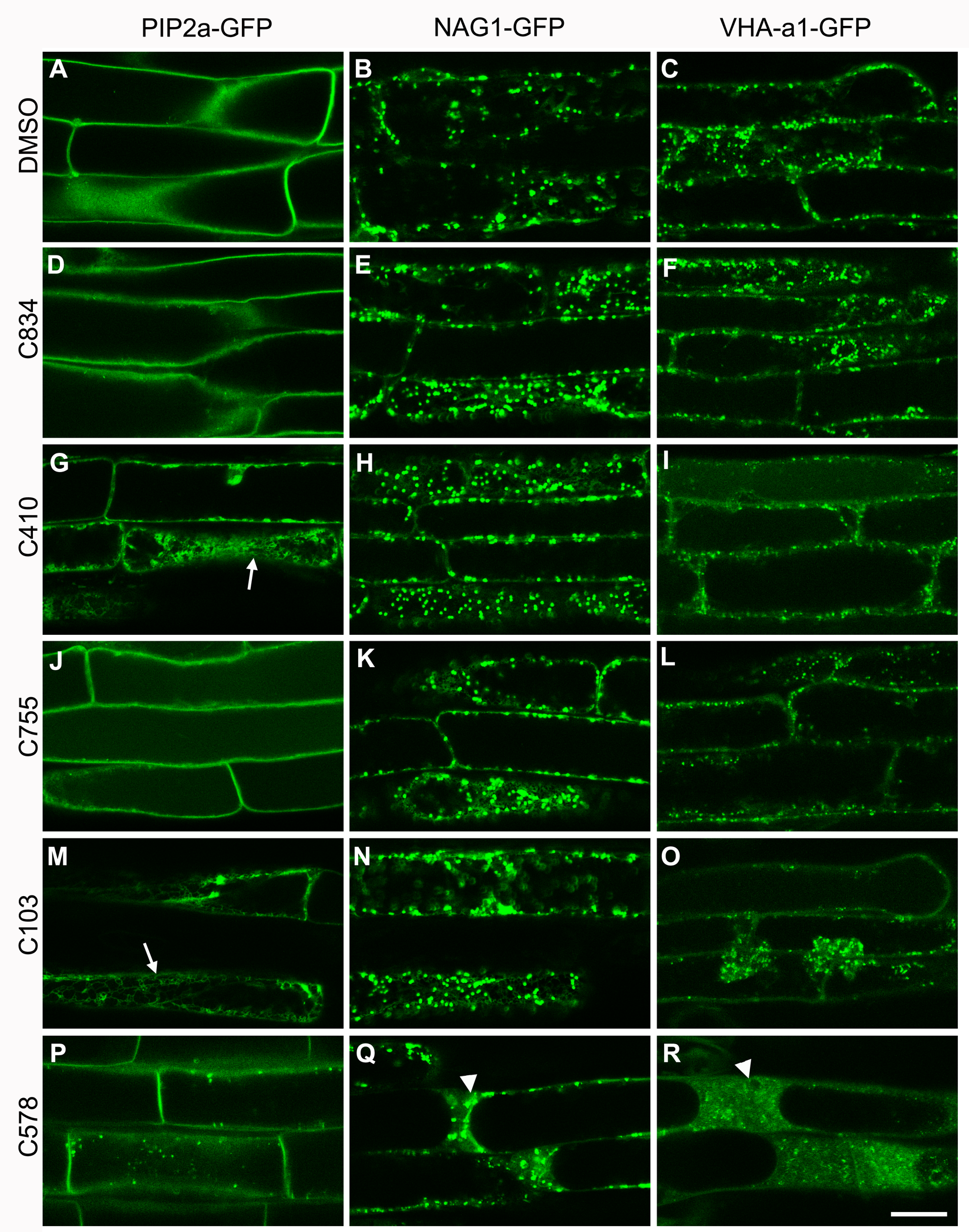

Supplement: Figure S3 — Effects of bioactive hits on endomembrane markers for Golgi, trans -Golgi network and plasma membrane. Three-day-old seedlings expressing PIP2A-GFP (A, D, G, J, M, P), NAG1-GFP (B, E, H, K, N, Q) and VHA-a1-GFP (C, F, I, L, O, R) were exposed to DMSO (control, A–C), 55 μM C834 (D–F), 62.34 μM C410 (G–I), 88 μM C755 (J–L), 79.14 μM C103 (M–O) or 80 μM C578 (P–R) for 48 h and imaged under a confocal microscope. ER network localization (arrows) was observed in C410 and C103-treated PIP2A-GFP. Cytoplasmic diffuse fluorescence induced by C578 is indicated with arrowheads. Bar = 20 μm. (TIF) [file pone.0044735.s003.tif]

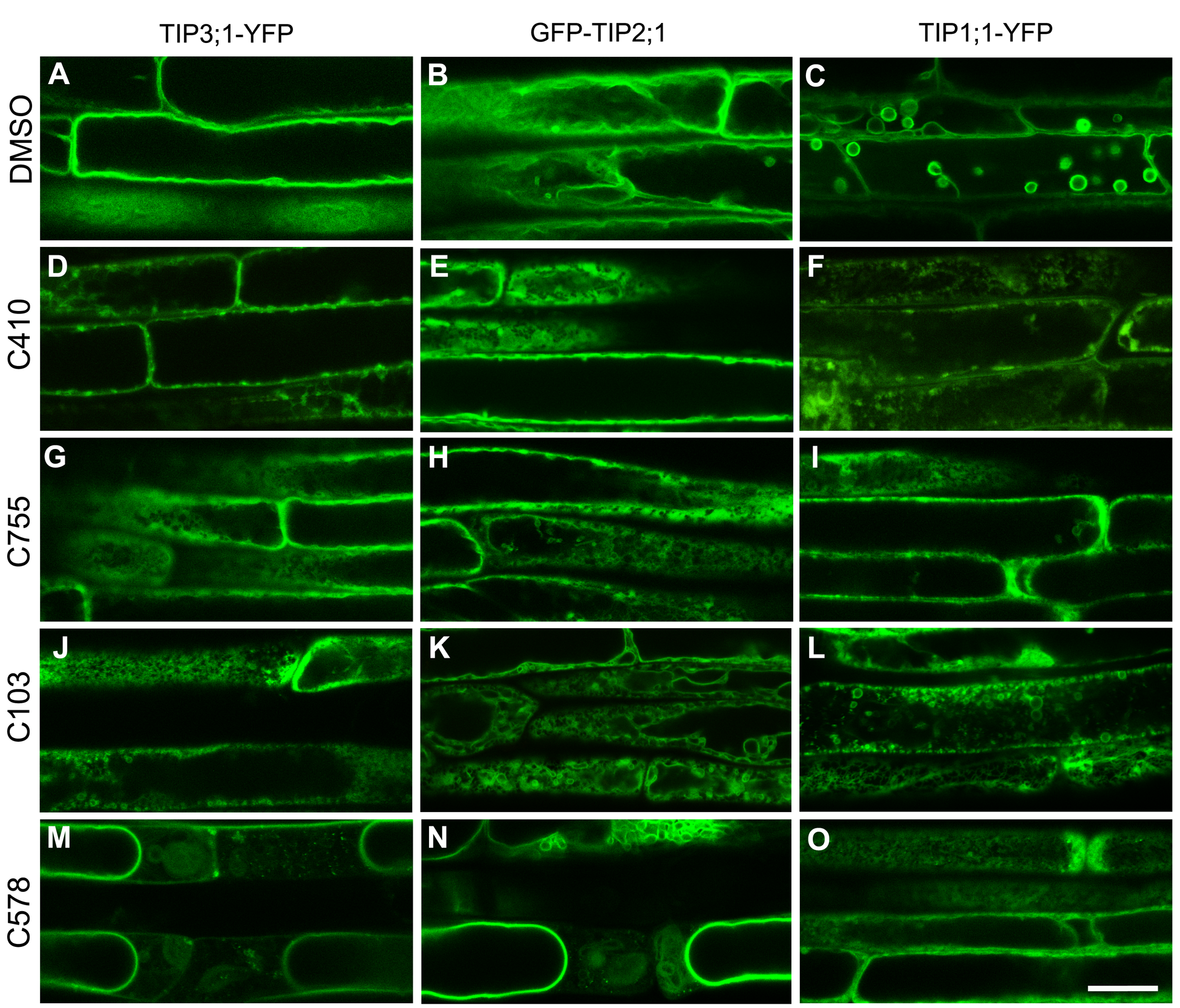

Supplement: Figure S4 — Class II and Class III probes disturb the trafficking of all three tonoplast intrinsic proteins. Three-day-old seedlings expressing, TIP3;1-YFP (A, D, G, J, M), GFP-TIP2;1 (B, E, H, K, N) or TIP1;1-YFP (C, F, I, L, O) were exposed to DMSO (control, A–C), 62.34 μM C410 (D–F), 88 μM C755 (G–I), 79.14 μM C103 (J–L) or 80 μM C578 (M–O) for 48 h and imaged under a confocal microscope. Bar = 20 μm. (TIF) [file pone.0044735.s004.tif]

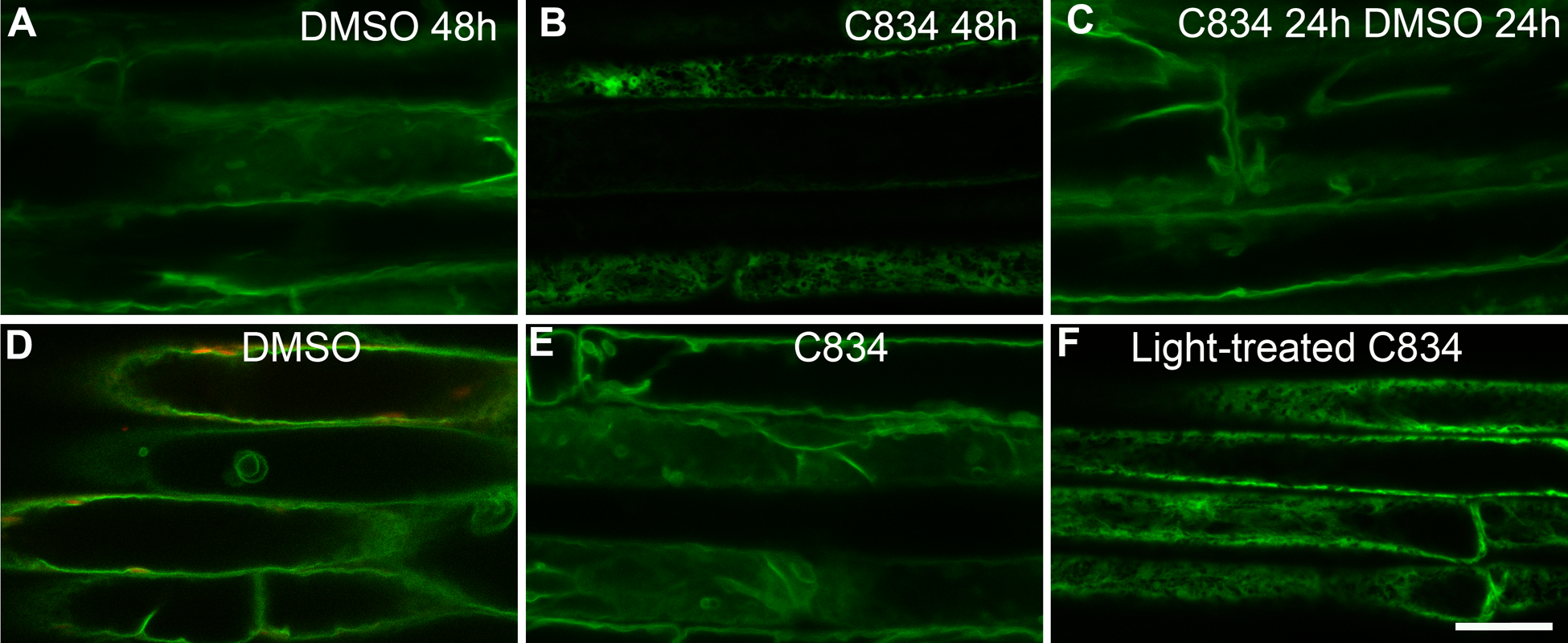

Supplement: Figure S5 — C834 bioactivity is reversible and requires light incubation. (A–C) Three-day old GFP-TIP2;1 seedlings were exposed to DMSO (A) or C834 (B) for 48 h, or exposed to C834 for 24 h and then transferred to liquid MS media for 24 h (C) before imaging. 24 h of C834 treatment induced the same phenotype as the one shown in (B). (D–F) Three-day-old GFP-TIP2;1 seedlings were transferred to media containing DMSO (D), 55 μM C834 (E) or 55 μM C834 media that was previously exposed to light for 16 h (F). Plates were incubated in the dark for 48 h before microscopic analysis. Only the light-treated C834 induces the ER localization of GFP-TIP2;1. Bar = 20 μm. (TIF) [file pone.0044735.s005.tif]

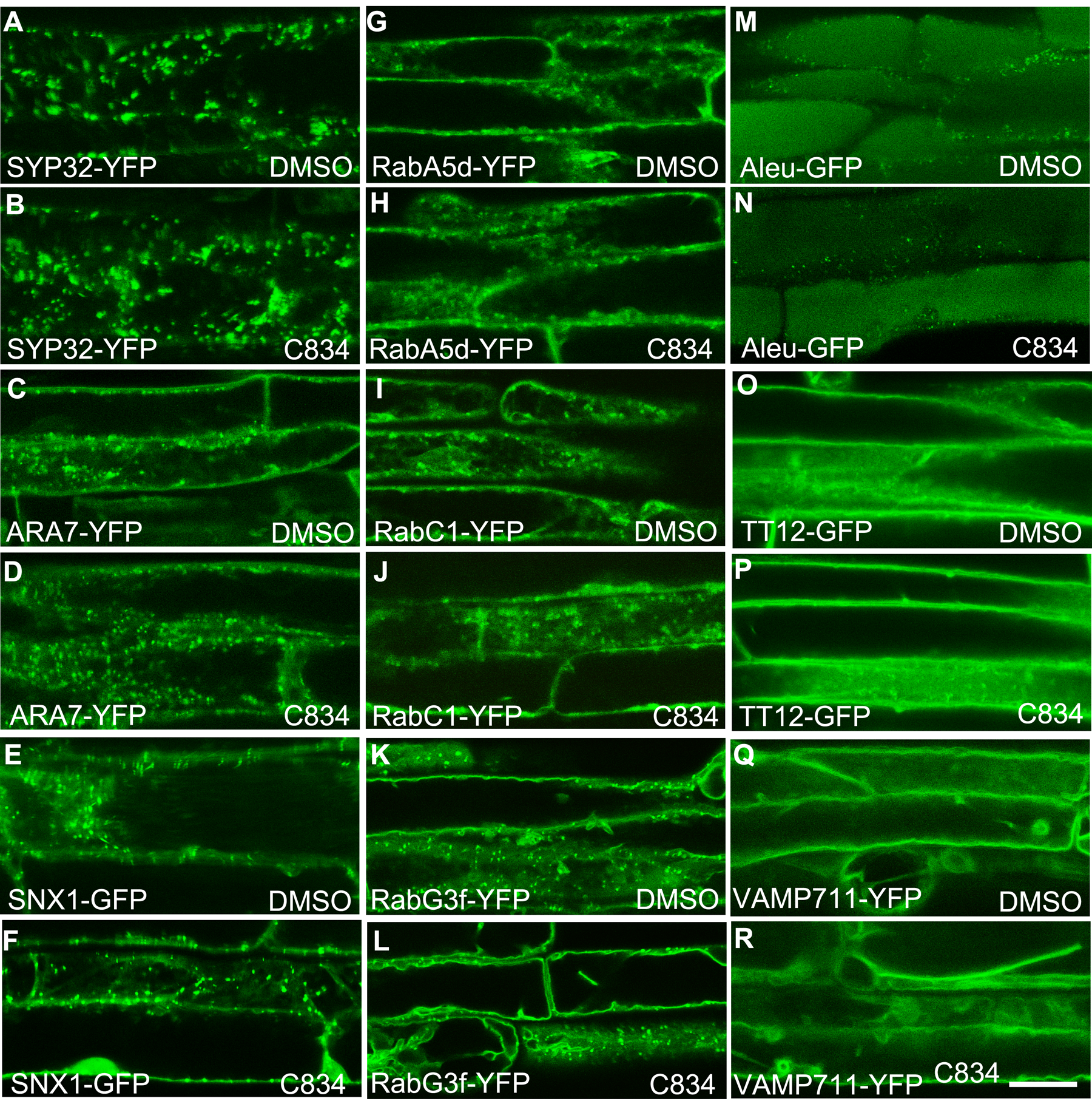

Supplement: Figure S6 — Effect of C834 on a diverse set of endomembrane markers. 3-day old seedlings expressing the indicated constructs were transferred to DMSO (A, C, E, G, I, K, M, O, Q) or C834 (B, D, F, H, J, L, N, P)-containing media for 48 h. Bar = 20 μm. (TIF) [file pone.0044735.s006.tif]

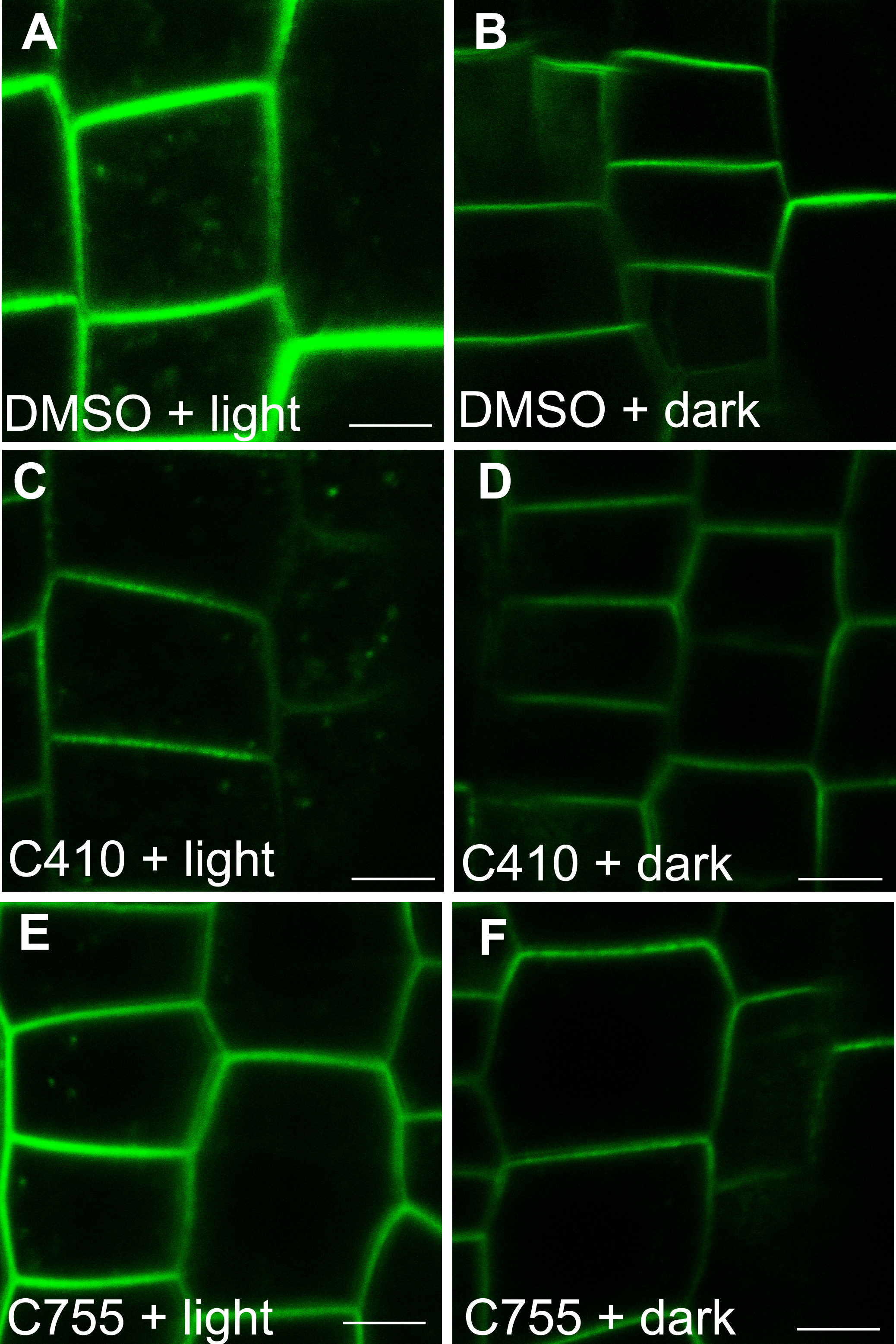

Supplement: Figure S7 — PIN2 trafficking in the dark is insensitive to Class II probes. Four-day-old light-grown seedlings expressing PIN2-GFP were transferred to either DMSO (control, A,B), 62.34 μM C410 (C, D), 88 μM C755 (E, F) for 18 h in the light (A, C, E) or the dark (B, D, F). All images of each marker were taken at the same microscope settings. Bar = 10 μm. (TIF) [file pone.0044735.s007.tif]
