# Supplementary material for: A Small Molecule Inhibitor Partitions Two Distinct Pathways for Trafficking of Tonoplast Intrinsic Proteins in Arabidopsis
Source: PLoS One. 2012 Sep 5;7(9):e44735. doi: 10.1371/journal.pone.0044735 (PMC3434187; doi:10.1371/journal.pone.0044735)
Supplement: Table S1 — Chemical structure, and Chembridge and PubChem ID numbers for the tonoplast trafficking inhibitors. Short name in parenthesis is used in the text. (PPT) [file pone.0044735.s010.ppt]

## Slide 1
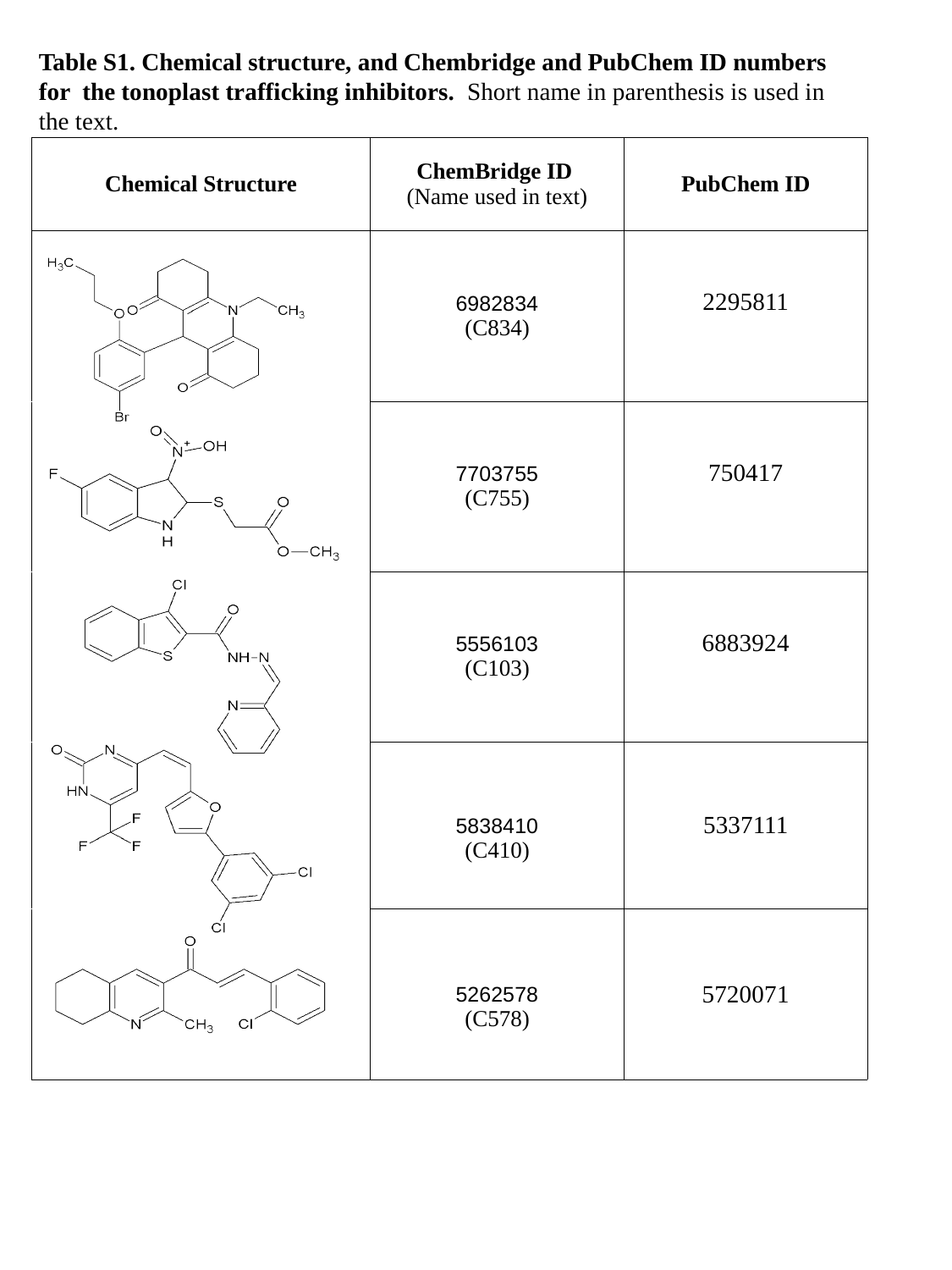

Table S1. Chemical structure, and Chembridge and PubChem ID numbers for the tonoplast trafficking inhibitors. Short name in parenthesis is used in the text.
| Chemical Structure | ChemBridge ID (Name used in text) | PubChem ID |
| --- | --- | --- |
| | 6982834 (C834) | 2295811 |
| | 7703755 (C755) | 750417 |
| | 5556103 (C103) | 6883924 |
| | 5838410 (C410) | 5337111 |
| | 5262578 (C578) | 5720071 |
